# Degradation of beechwood xylan using food-grade bacteria-like particles displaying β-xylosidase from *Limosilactobacillus fermentum*

**DOI:** 10.1186/s40643-025-00898-1

**Published:** 2025-06-19

**Authors:** Robie Vasquez, Ji Hoon Song, Jae Seung Lee, Bernadette Bagon, Sanghoon Kim, Valerie Diane Valeriano, Dae-Kyung Kang

**Affiliations:** 1 Department of Animal Biotechnology, Dankook University, Cheonan, 31116 Republic of Korea; 2 Department of Microbiology, Tumor and Cell Biology, Centre for Translational Microbiome Research (CTMR), Karolinska Institutet, Stockholm, 17165 Sweden; 3 Present Address: College of Pharmacy and Pharmaceutical Sciences, Florida A&M University, Tallahassee, FL 32301 USA

**Keywords:** Surface display, Beta-xylosidase, Lactic acid bacteria, Xylan, Immobilisation, Biocatalyst

## Abstract

The display of enzymes on bacterial surfaces is an interesting approach for immobilising industrially important biocatalysts. In recent years, non-recombinant surface display using food-grade bacteria, such as lactic acid bacteria (LAB), have gained interest because of their safety, simplicity, and cost-effectiveness. β-Xylosidase is one of the many biocatalytic enzymes targeted for immobilisation due to its key role in the complete saccharification of lignocellulosic biomass, including xylan hemicellulose. Recently, the xylose-tolerant β-xylosidase, LfXyl43, was identified in *Limosilactobacillus fermentum.* LfXyl43 is capable of producing xylose from the degradation of xylo-oligosaccharides (XOS) and beechwood xylan. This study aimed to immobilise this new biocatalyst on the surface of LAB-derived bacteria-like particles (BLP) and investigate its applicability and reusability in the degradation of xylan hemicellulose. Additionally, the influence of the anchor position and the presence of linker peptides on the display and activity of the β-xylosidase was investigated. Four expression vectors were constructed to express different anchor-xylosidase fusion proteins. Upon expression and purification, all anchor-xylosidase fusion proteins were active towards the artificial substrate *p*-nitrophenyl-β-D-xylopyranoside. In addition, all anchor-xylosidase fusion proteins were successfully displayed on the surface of BLP. However, only the β-xylosidases with linker peptide showed hydrolytic activity after immobilisation on BLP. BLP displaying β-xylosidases demonstrated high activity against XOS and beechwood xylan, thereby producing high amounts of xylose. Moreover, the immobilised enzyme demonstrated reusability across several bioconversion cycles. Overall, this study highlights the potential industrial application of surface-displayed β-xylosidase for the effective degradation of lignocellulosic biomass.

## Graphical abstract

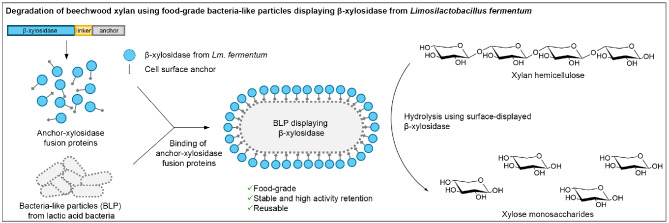

## Introduction

β-Xylosidase (1,4-β-D-xylan xylohydrolase, E.C. 3.2.1.37) is an economically important enzyme for the complete and efficient saccharification of lignocellulosic biomass, particularly xylan hemicellulose (Rohman et al. 2019; Li et al. 2023). The hydrolysis of xylan hemicellulose into xylose monosaccharides is a cost-effective and sustainable alternative source of biofuels, chemicals, and many bio-based products (Saha 2003; Naidu et al. 2018). The complete degradation of xylan hemicellulose is a result of concerted hydrolytic activities of different enzymes, including β-xylosidase, which hydrolyses the glycosidic bonds of xylo-oligosaccharides (XOS), producing high amounts of xylose (Lagaert et al. 2014; Rohman et al. 2019; Mohammadi Moradian et al. 2024). Owing to its crucial role in the complete conversion of lignocellulosic biomass, β-xylosidases have attracted significant interest (Saha 2003). Numerous β-xylosidases have been identified and characterised from fungal, bacterial, and even metagenomic sources, with many demonstrating promising potential for industrial applications (Lagaert et al. 2014; Bosetto et al. 2016; Rohman et al. 2019; Li et al. 2023). This diversity and ubiquity give rise to different classes of β-xylosidases, with different biochemical properties and substrate specificities. Lactic acid bacteria (LAB) are excellent sources of glycoside hydrolase (GH) enzymes, including β-xylosidases, owing to their diversity and niche adaptations (Michlmayr and Kneifel 2014; Vasquez et al. 2024). LAB, such as *Lactobacillus*,* Leuconostoc*,* Pediococcus*,* Streptococcus*,* Weissella*, and *Oenococcus* can utilise XOS and xylan due to their β-xylosidase activity (Ohara et al. 2006; Moura et al. 2007; Jang and Kim 2011; Maria et al. 2014; Iliev et al. 2020). In their work, Michlmayr et al. (2013) characterised two catalytically active β-xylosidases from *Lactobacillus brevis* DSM 20054. Meanwhile, Falck et al. (2016) characterised a β-xylosidase from *Weissella* sp. 92, with hydrolytic activity towards XOS. Another β-xylosidase was isolated from *Lactobacillus rossiae* DSM 15824^T^ and has potential applications in sourdough production (Pontonio et al. 2016). Recently, we characterised a β-xylosidase from *Limosilactobacillus fermentum* SK152 belonging to GH family 43 subfamily 11 (Vasquez et al. 2025a). This β-xylosidase, termed LfXyl43, showed high hydrolytic activity towards XOS and beechwood xylan. Interestingly, LfXyl43 also exhibited exceptional tolerance to xylose–a characteristic rarely reported in β-xylosidases found in LAB. These properties make LfXyl43 a promising biocatalyst candidate for industrial processes.

Several recent studies have explored the immobilisation of hydrolytic enzymes on the surfaces of various support materials. Enzyme immobilisation can be achieved through various techniques, such as adsorption, covalent bonding, entrapment, and cross-linking (Sirisha et al. 2016; Maghraby et al. 2023; Lu et al. 2025). In industrial applications, immobilised enzymes have several advantages over non-immobilised enzymes, such as improvement of enzyme stability, shelf-life, and efficiency (Garcia-Galan et al. 2011; Homaei et al. 2013; Maghraby et al. 2023; Li et al. 2025). Compared to free enzymes, immobilised biocatalysts offer improved cost-effectivity and enhance the sustainability of the bioconversion process owing to their reusability and efficiency (Maghraby et al. 2023; Ali et al. 2024; Li et al. 2025). The use of immobilised enzymes has also been explored in different biomedical applications but has been hampered by problems arising from stability, potential toxicity, and allergic reactions (Homaei et al. 2013). Hence, the interest in the use of food-grade carriers for enzyme immobilisation studies has increased. In addition to their probiotic applications, LAB have demonstrated great potential as food-grade carriers for biocatalytic enzymes owing to their Generally regarded as safe (GRAS) status (Mathiesen et al. 2020). Immobilisation of enzymes on LAB-based carriers can be achieved through surface display approaches, either recombinant or non-recombinant (Zadravec et al. 2015; Michon et al. 2016; Mao et al. 2016). In the former approach, a recombinant LAB expressing and displaying the desired biocatalyst is developed, whereas in the latter, the desired biocatalyst is produced separately and displayed on non-genetically modified organisms (non-GMOs), such as LAB (Lee et al. 2003; Zadravec et al. 2015). While the recombinant approach is advantageous when continuous production and display of the enzyme is desired, a non-GMO surface display is a suitable alternative when market acceptability is of great concern. Although still in their infancy, pioneering studies have begun to investigate the use of non-GMO LAB for the immobilisation of industrially important biocatalysts.

Therefore, this study aimed to develop a food-grade immobilised biocatalyst for the degradation of XOS and xylan through the non-GMO surface display of the xylose-tolerant β-xylosidase LfXyl43. In this study, a non-classical surface anchor from LAB, CshA, was utilised to display the β-xylosidase on LAB-derived bacteria-like particles (BLP). The influence of the position of the surface anchor and the presence of linker peptides on enzyme activity was also examined. Finally, the activity of the surface-displayed β-xylosidase towards XOS and xylan and its reusability were investigated. The findings of this study provide a cost-effective and eco-friendly food-grade alternative for industrial lignocellulose biomass degradation.

## Materials and methods

### Bacterial strains, plasmid, primers, and culture conditions

The bacterial strains and plasmids used in this study are summarised in Table 1. *Lm. fermentum* SK152, isolated from kimchi by Yoo et al. (2017), was cultured in Man Rogosa Sharpe (MRS) broth (BD Difco, NJ, USA) at 37 °C without aeration. *Escherichia coli* DH5α and *E. coli* BL21 (DE3) competent cells were used for molecular cloning and protein expression, respectively. *E. coli* strains were grown in Luria-Bertani (LB) broth (BD Difco, NJ, USA) supplemented with ampicillin (100 µg/mL) at 37 °C with aeration. The pET21b (+) plasmid vector was used to construct a 6× histidine-tagged anchor-xylosidase fusion expression vector.

**Table 1:** Bacterial strains and plasmids used in this study

Strains/Plasmids	Features	Source
*Escherichia coli* DH5α	Cloning host	Biofact
*E. coli* BL21 (DE3)	Expression host	Real BioTech
*Limosilactobacillus fermentum* SK152	Source of β-xylosidase gene, *xyl*; source of bacteria-like particles	(Yoo et al. 2017)
*Lactiplantibacillus plantarum* SK156	Source of the surface anchor gene, *cshA*	(Hwang et al. 2021)
pET21b (+)	Expression vector with 6× histidine tag, Amp^r^, T7 promoter	Novagen
pET21-CshA-Xyl	pET21b (+) carrying histidine-tagged *cshA*-*xyl* fusion gene	This study
pET21-Xyl-CshA	pET21b (+) carrying histidine-tagged *xyl-cshA* fusion gene	This study
pET21-CshA-L-Xyl	pET21b (+) carrying histidine-tagged *cshA-l-xyl* fusion gene with (GGGGS)_3_ linker	This study
pET21-Xyl-L-CshA	pET21b (+) carrying histidine-tagged *xyl-l-cshA* fusion gene with (GGGGS)_3_ linker	This study

### Molecular cloning

Table 2 lists the primers used in this study. The primers were designed to contain overlapping sequences for fusion PCR and enzyme sites for *Nde*I and *Xho*I. *Taq* polymerase (TaKaRa, Tokyo, Japan) was used for all PCR in this study. The genomic DNA of *Lm. fermentum* SK152 was extracted according to Yoo et al. (2017) and was used as the template to amplify the β-xylosidase gene *xyl* (GenBank accession No. PQ818275), using the primer pairs X1f and X1r, X2f and X2r, X3f and X3r, and X4f and X4r. The *cshA* gene from *Lactiplantibacillus plantarum* SK156 was amplified following our previous protocol (Vasquez et al. 2022) using the primer pairs C1f and C1r, C2f and C2r, C3f and C3r, and C4f and C4r. To construct the templates for the flexible linker peptide containing three repeats of GGGGS residues [(GGGGS)_3_], sense (L1f and L2f) and antisense (L1r and L2r) sequences were purchased from a third-party company (Bionics, Republic of Korea) and were annealed via touchdown PCR (annealing: 95 °C, − 5 °C/cycle, 30 s, 15 cycles; extension: 72 °C, 30 s). To construct the fusion gene fragments without the linker sequence, touchdown PCR was performed using the primers C1f and X1r for the N-terminal anchor construct and the primers X2f and C2r for the C-terminal anchor construct. To construct the N-terminal anchor fusion with the (GGGGS)_3_ linker, the primers C3f and L1r were used to fuse the anchor-linker sequences, the primers L1f and X3r were used to fuse the linker-xylosidase sequences, and the primers C3f and X3r were used to create the anchor-xylosidase fusion gene with the linker. To construct the C-terminal anchor fusion with the (GGGGS)_3_ linker, the primers X4f and L2r were used to combine the xylosidase-linker sequences, whereas the primers L2f and C4r were used to combine the linker-anchor sequences; the primers X4f and C4r were used to create the xylosidase-anchor fusion gene with the linker. The amplicons were run through a 1% (w/v) agarose gel, excised, and cleaned using a commercial kit (Machery-Nagel, Düren, Germany). The expression vectors pET21-CshA-Xyl, pET21-Xyl-CshA, pET21-CshA-L-Xyl, and pET21-Xyl-L-CshA were constructed by double digestion of the respective fusion fragments using the restriction enzymes *Nde*I and *Xho*I (TaKaRa, Tokyo, Japan) and then ligated into the pET21b (+) vector (Novagen, Merck Millipore, MA, USA) using T4 ligase (TaKaRa, Tokyo, Japan). Sequence correctness was verified by transforming the expression vectors into *E. coli* DH5α competent cells.

**Table 2:** Nucleotide sequences of primers and linkers used in this study

Primers	Sequence (5’ → 3’)
C1f	AAA GAA TTC **CAT ATG** GCG AGT GAG AAG CTC
C1r	*TTG GAT AGT TTT CAT* CGT TTC ACC ATC ACC TTT
X1f	*AAA GGT GAT GGT GAA* ACG ATG AAA ACT ATC CAA
X1r	AAA AAA AAG CTT **CTC GAG** CCG GCT
X2f	GGG GAA TTC **CAT ATG** AAA ACT ATC CAA AAT CCG ATT ATT CCG GG
X2r	*CTT CTC ACT CGC* CCG GCT CGT TAC
C2f	*GTA ACG AGC CGG* GCG AGT GAG AAG
C2r	AAA AAG CTT **CTC GAG** CGT TTC ACC ATC ACC TTT GTA G
L1f	*GGT GAT GGT GAA ACG* GGC GGC GGC GGC TCC GGC GGC GGT GGA AGT GGA GGG GGC GGG TCG *ATG AAA ACT ATC CAA*
L1r	*TTG GAT AGT TTT CAT* CGA CCC GCC CCC TCC ACT TCC ACC GCC GCC GGA GCC GCC GCC GCC *CGT TTC ACC ATC ACC*
C3f	GGG GAA TTC **CAT ATG** GCG AGT GAG AAG CTC
C3r	*CC GCC GCC* CGT TTC ACC
L1r	TTG GAT AGT TTT CAT *CGA CCC GCC CCC*
L1f	*GGT GAT GGT GAA ACG* GGC GGC
X3f	*GGG GGC GGG TCG* ATG AAA ACT ATC CAA
X3r	AAA AAG CTT **CTC GAG** CCG GCT CGT TAC C
L2f	*CGG GTA ACG AGC CGG* GGC GGC GGC GGC TCC GGC GGC GGT GGA AGT GGA GGG GGC GGG TCG *GCG AGT GAG AAG CTC*
L2r	*GAG CTT CTC ACT CGC* CGA CCC GCC CCC TCC ACT TCC ACC GCC GCC GGA GCC GCC GCC GCC *CCG GCT CGT TAC CCG*
X4f	GGG GAA TTC **CAT ATG** AAA ACT ATC CAA AAT CCG ATT ATT CCG GG
X4r	*CC GCC* CCG GCT CGT TAC
L4r	*GAG CTT CTC ACT CGC* CGA CCC
L4f	*CGG GTA ACG AGC CGG* GG
C4f	*GGG TCG* GCG AGT GAG AAG CTC
C4r	AAA AAG CTT **CTC GAG** CGT TTC ACC ATC ACC TTT GTA G

Enzyme restriction sites *Nde*I (CAT ATG) and *Xho*I (CTC GAG) are in bold

Overlapping sequences for fusion PCR are in italics

### Protein expression and purification

To overexpress the fusion proteins, each expression vector was transformed into *E. coli* BL21 (DE3) competent cells. Then, the *E. coli* BL21 cells harbouring either pET21-CshA-Xyl, pET21-Xyl-CshA, pET21-CshA-L-Xyl, or pET21-Xyl-L-CshA were grown overnight in LB broth with 100 µg/mL ampicillin (final concentration). From the overnight culture, 1 mL was then added to 100 mL of LB broth supplemented with ampicillin and incubated until it reached an optical density of 0.5–0.6 at 600 nm. The expression of the protein was induced by adding 0.1 mM (final concentration) of isopropyl-β-D-thiogalactopyranoside (IPTG) to the culture. Overexpression was performed at 20 °C for 12 h, with gentle shaking. After the incubation period, cells were harvested following centrifugation at 10,000 × *g*, 4 °C, for 10 min. The cell pellets were washed twice with 50 mM sodium phosphate buffer (pH 7.0) containing 1 mM phenylmethylsulphonyl fluoride (PMSF). Thereafter, the cells were resuspended in 50 mM sodium phosphate buffer (with 1 mM PMSF and 10 mM imidazole, pH 7.0) and disrupted using a sonicator (10 cycles of 10 s sonication and 10 s pause, on ice). The cell-free extract was collected by centrifugation (15,000 × *g*, 4 °C, 15 min) and filtered using a 0.22-µm filter. The 6× histidine-tagged fusion proteins were purified using nickel-nitrolotriacetic acid (Ni-NTA) agarose resin (Qiagen, Hilden, Germany) and eluted with 50 mM sodium phosphate buffer containing 250 mM imidazole. To remove the imidazole after the purification step, the fusion proteins were dialysed against 50 mM sodium phosphate buffer containing 20% (v/v) glycerol (pH 7.0) using a dialysis membrane (30,000 MWCO) at 4 °C overnight. Protein concentrations were determined using a protein assay dye (Bio-Rad, Hercules, CA, USA). Bovine serum albumin (BSA; Bio-Rad, Hercules, CA, USA) was used as the standard. Purified fusion proteins were loaded onto 12% SDS-PAGE gels to verify the protein size.

### Surface display assay

The preparation, quality control, and storage of BLP derived from *Lm. fermetum* was performed according to our methods as described previously (Vasquez et al. 2022, 2025b). The display of the fusion proteins on the surface of BLP was performed as described in Vasquez et al. (2022) with some modifications. Briefly, 1 × 10^9^ BLP were incubated with 100 µg of the fusion protein (2 U) in 50 mM sodium phosphate buffer (pH 7.0) at 37 °C for 2 h. After the incubation period, the BLP were collected by centrifugation at 5,000 × *g*, 4 °C, for 3 min. The supernatants containing the unbound fusion proteins were collected and stored at − 20 °C until further use. The BLP displaying the fusion proteins were washed thrice with 50 mM sodium phosphate buffer (pH 7.0). The BLP displaying the β-xylosidase were resuspended in 50 mM sodium phosphate buffer (pH 7.0) and kept at − 20 °C until assayed.

The immobilisation parameters were calculated according to Boudrant et al. (2020). The residual activity of the unbound enzyme was defined as the percentage of the activity of the unbound enzyme after surface display relative to the activity of the free enzyme (total applied activity). The surface display (immobilisation) yield was calculated by subtracting the activity of the unbound enzyme from the total applied activity. The activity on the cell surface (%) was calculated as the ratio of the activity of the surface-displayed enzyme to the total applied activity; the activity on the cell surface was calculated based on units of enzymes (U) per dry weight of BLP (g). Finally, activity retention (%) was calculated as the ratio of the activity of the surface-displayed enzyme (%) to the surface display yield (%).

### Immunofluorescence microscopy and flow cytometry

Immunofluorescence microscopy and flow cytometry were performed using previously described methods, with some modifications (Hwang et al. 2023; Vasquez et al. 2025b). Briefly, non-displaying BLP or BLP displaying the β-xylosidase were collected and resuspended in 1× phosphate-buffered saline (PBS) with 0.1% (v/v) Tween 20 and 2% (w/v) BSA (PBST-B) and anti-His antibody (R&D Systems, MN, USA; 1:300 dilution), then incubated overnight at 4 °C. Thereafter, the BLP were collected by centrifugation and washed five times with PBST-B. Then, the BLP were resuspended in PBST-B with donkey anti-mouse IgG NorthernLights™ NL557-conjugated antibody (R&D Systems, MN, USA; 1:300 dilution) and incubated in the dark for 1 h at room temperature. Subsequently, the BLP were washed five times with PBS. The surface display was visualised under a fluorescence microscope (ProgRes C10 plus with Intensilight C-HGFI; Nikon, Tokyo, Japan) equipped with a 570 nm filter. The population of BLP displaying β-xylosidase was determined using flow cytometry (BD Accuri™ C6 Plus; BD Biosciences, NJ, USA). Non-displaying BLP were used as controls.

### Enzyme assay

The enzyme assays in this study were performed by adding the free fusion proteins or approximately 1 × 10^9^ BLP displaying the β-xylosidase into 50 mM sodium phosphate buffer (pH 7.0) with 2 mM *p*-nitrophenyl-β-D-xylopyranoside (pNPX; Sigma, MO, USA) or 2 mM XOS (xylobiose, xylotriose, or xylotetraose; Megazyme, Ireland) at 35 °C for 5 min. After the assay, the BLP were collected by centrifugation (5,000 × *g*, 5 min, 4 °C). For the pNPX assay, an equal volume of 1 M Na_2_CO_3_ was added to the supernatant to stop the reaction. The enzyme activity was measured by the release of *p*-nitrophenol, spectrophotometrically detected at 405 nm (SpectraMax; Molecular Diagnostics, CA, USA). For XOS substrates, an equal volume of 3, 5-dinitrosalycylic acid (DNSA) was added to the supernatant and boiled for 5 min (Miller 1959). After cooling, the release of reducing sugars equivalent to xylose was spectrophotometrically measured at 540 nm. The amount of enzyme needed to release 1 µmol of *p*-nitrophenol or 1 µmol of reducing sugars per minute under the assay conditions was defined as one unit (U) enzyme activity. All assays were performed in triplicate.

The reusability of surface-displayed β-xylosidase was determined by repeatedly performing the enzyme assay described above using pNPX as the substrate. After each round, the BLP were harvested and washed with 50 mM sodium phosphate buffer (pH 7.0) before adding a fresh substrate solution. The residual activity was measured by comparing the activity to that of the first round of the assay, which was set at 100%.

### Thin-layer chromatography

Five microlitres of the reaction supernatant were spotted on silica gel F_254_ plates (Merck, MA, USA) and double-ascended using a mobile phase consisting of 1-butanol: acetic acid: water (2:1:1, v/v/v). The bands were visualised by spraying diphenylamine: aniline: acetone: phosphoric acid solution (2:2:100:15, w/v/v/v) onto the plates followed by heating.

### Time course of xylose production and high-performance liquid chromatography

The production of xylose via the degradation of beechwood xylan by surface-displayed β-xylosidase was monitored using high-performance liquid chromatography (HPLC), following the methods of Corradini et al. (2021). Briefly, the reaction contained 30 g/L beechwood xylan (Serva, Heidelberg, Germany) in 50 mM sodium phosphate buffer (pH 7.0) and BLP displaying either CshA-L-Xyl or Xyl-L-CshA at a final concentration of 0.5 mg displayed β-xylosidase per mL of reaction volume. The reaction took place at 35 °C with shaking for 24 h. Afterwards, the samples were centrifuged (10,000 × *g*, 5 min, 4 °C), then the supernatant was filtered using a 0.20-µm PTFE membrane filter before injection into an Agilent 1260 Infinity (Agilent, CA, USA) HPLC system. The HPLC was equipped with a Waters Sugar-Pak™ I column (300 × 6.5 mm, 10 μm; Waters, MA, USA) maintained at 80 °C and connected to a refraction index detector (45 °C). Ultrapure water was used as the mobile phase, and the flow rate was 0.5 mL/min. Xylose was used as the standard for the calibration curve.

### Statistical analysis

All data are reported as mean ± standard deviation (SD) of triplicate experiments. All SDs were less than 5%.

## Results

### Molecular cloning and expression of fusion proteins

**Fig. 1:**
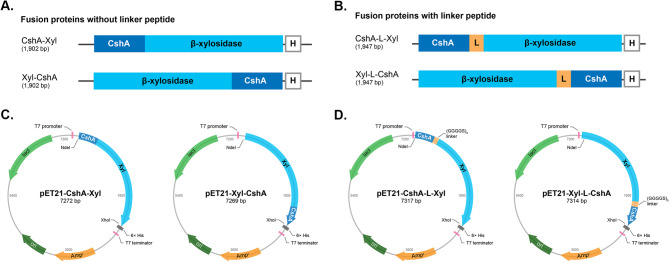
Construction of the anchor-xylosidase fusion protein expression vectors. Fusion of the anchor, CshA, to the β-xylosidase gene, at either the N- or C-terminus, with or without the (GGGGS)_3_ peptide linker (**A-B**). The linker peptide is denoted by “L”, whereas the 6× histidine tag is denoted by “H”. Expression vectors of the histidine-tagged fusion proteins with and without the linker peptide based on the pET21b (+) vector (**C-D**)

**Fig. 2:**
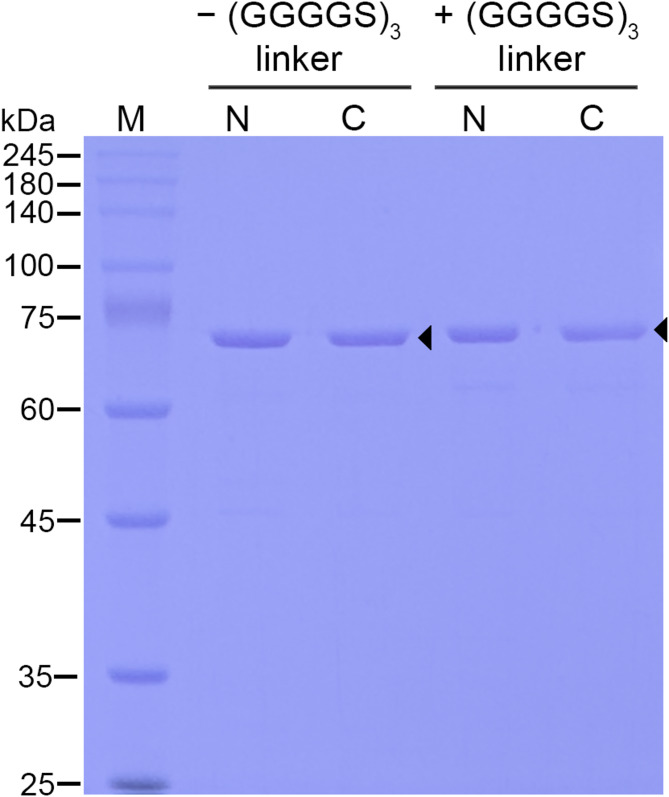
SDS-PAGE analysis of the Ni-NTA purified fusion proteins with and without the flexible (GGGGS)_3_ linker peptide. N and C denote the N- or C-terminal position of the cell surface anchor CshA, respectively. The expected protein size of the fusion proteins without the linker peptide (CshA-Xyl and Xyl-CshA) is 71.2 kDa, whereas that of the fusion proteins with linker peptide (CshA-L-Xyl and Xyl-L-CshA) is 72.1 kDa. M indicates the protein markers (LPS Solution, Daejeon, Republic of Korea). Arrows indicate the expected sizes of the fusion proteins

To construct fusion proteins containing both the cell surface anchor, CshA, and the β-xylosidase, CshA was fused either at the N- or C- terminus of the β-xylosidase via PCR (Fig. 1A). Fusion proteins with three repeats of the flexible GGGGS linker peptide between the anchor and enzyme were also constructed via PCR (Fig. 1B). Four fusion protein constructs were cloned into the expression vector pET21b (+) via *Nde*I and *Xho*I restriction sites to create the following fusion protein expression vectors: pET21-CshA-Xyl, pET21-Xyl-CshA, pET21-CshA-L-Xyl, and pET21-Xyl-L-CshA (Fig. 1C and D). The expression vectors were transformed into *E. coli* BL21 (DE3) to overexpress the fusion proteins. After expression and purification, the fusion proteins were evaluated using SDS-PAGE (Fig. 2). SDS-PAGE analysis showed a single prominent band for each fusion protein immediately below the 75 kDa marker. The expected size of the fusion proteins without the linker peptide was 71.2 kDa, whereas that of the fusion proteins with the (GGGGS)_3_ linker peptide was 72.1 kDa. As expected, the molecular weight of the latter was higher due to the addition of the linker peptide (0.96 kDa). This indicated that all fusion proteins were expressed correctly. To test the β-xylosidase activity of the fusion proteins, enzyme assays using pNPX as the substrate were performed. Table 3 shows the specific activities (U/mg) of the free fusion proteins towards pNPX. All free fusion proteins were active towards the substrates without discernible differences in their hydrolytic activities. These results suggest that the location of the anchor and the presence of the linker peptide do not affect the activity of these free fusion proteins.

**Table 3:** Enzyme activities of purified anchor-xylosidase fusion proteins towards 2 mM *p*-nitrophenyl-β-D-xylopyranoside

Fusion protein	Specific activity, U/mg
CshA-Xyl	6.4 ± 0.1
Xyl-CshA	6.5 ± 0.1
CshA-L-Xyl	6.6 ± 0.2
Xyl-L-CshA	6.7 ± 0.1

The enzyme assays were performed in 50 mM sodium phosphate buffer (pH 7.0), at 35 °C, for 5 min. Values are reported as mean ± standard deviation of triplicate experiments

### Surface display of β-xylosidase on the surface of BLP

**Fig. 3:**
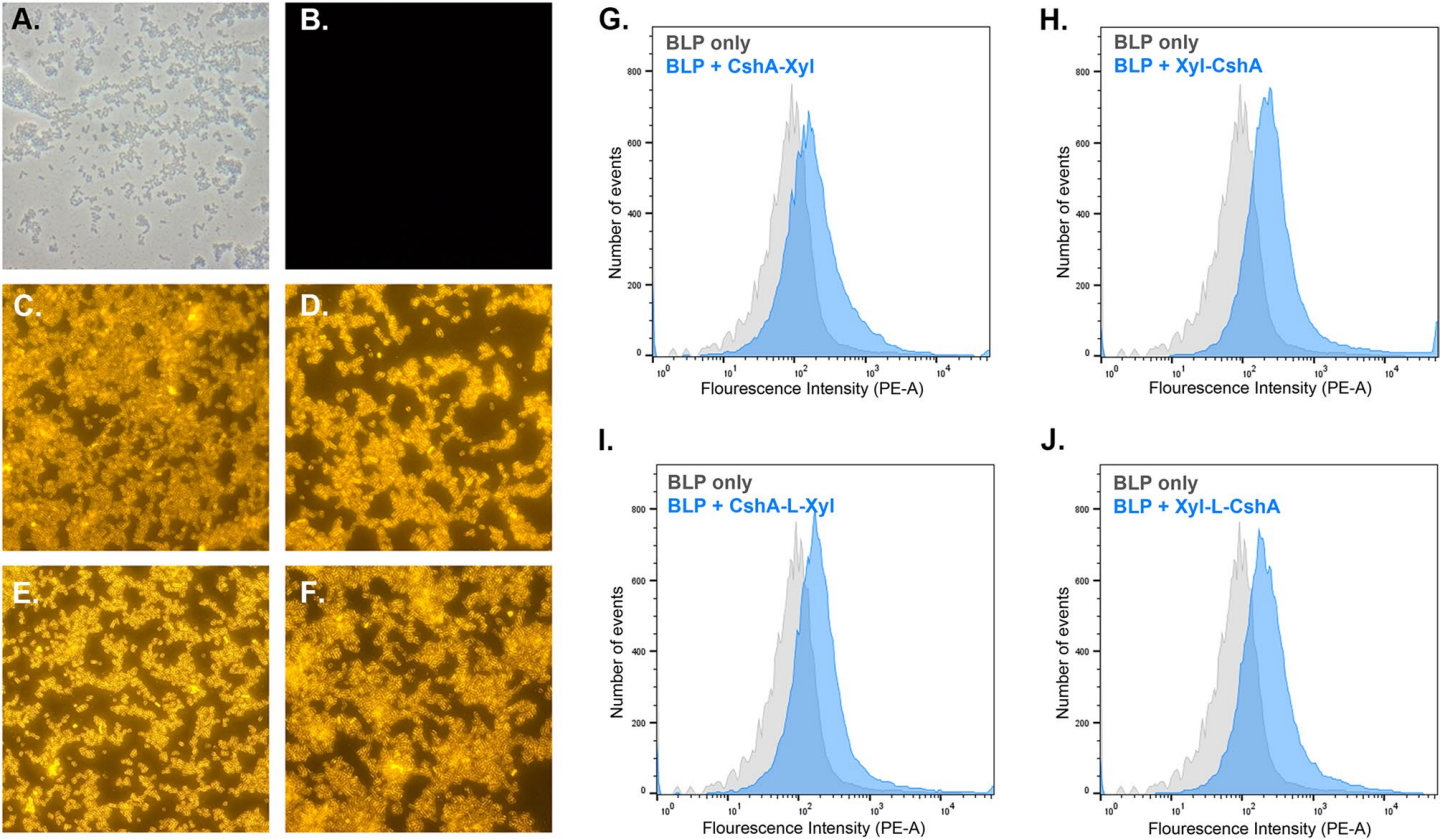
Surface display of β-xylosidase on lactic acid bacteria-derived bacteria-like particles (BLP) detected by immunofluorescence microscopy and flow cytometry. BLP used as a control for immunofluorescence microscopy (**A**). Non-displaying BLP (**B**), BLP + CshA-Xyl (**C**), BLP + Xyl-CshA (**D**), BLP + CshA-L-Xyl (**E**), and BLP + Xyl-L-CshA (**F**) viewed with a 570 nm filter. Flow cytometry measurements of the fluorescence intensity of BLP displaying β-xylosidase than that of non-displaying BLP: BLP + CshA-Xyl (**G**), BLP + Xyl-CshA (**H**), BLP + CshA-L-Xyl (**I**), and BLP + Xyl-L-CshA (**J**)

To display the β-xylosidase on the surface of LAB-derived BLP, each fusion protein was allowed to bind to the surface of BLP. The maximum binding capacity of the anchor CshA on 1 × 10^9^ BLP is 2.15 µM (approximately 80 µg fusion protein), as determined previously (Vasquez et al. 2022). Herein, 100 µg of fusion protein was added per 1 × 10^9^ particles and incubated at 37 °C for 2 h. To assess the surface display, immunofluorescence microscopy and flow cytometry experiments were performed. Immunofluorescence microscopy revealed that non-displaying BLP showed no fluorescence (Fig. 3A and B). By contrast, BLP displaying β-xylosidase showed good fluorescence indicating that the β-xylosidase was properly displayed by the CshA anchor (Fig. 3C–F). This also suggests that the CshA-mediated surface display of β-xylosidase was not affected by the anchor position or the presence of a linker peptide. These immunofluorescence microscopy results were corroborated by flow cytometry results (Fig. 3G–J). Compared to non-displaying BLP, all BLP displaying β-xylosidase exhibited a shift in the fluorescence intensity. These results indicate that β-xylosidase was successfully displayed on the surface of BLP.

To determine the functionality of surface-displayed β-xylosidase, enzyme assays were performed. Table 4 shows the immobilisation parameters calculated for each surface-displayed β-xylosidase. Unbound fusion proteins (supernatant after the surface display assay) showed residual activity ranging from 44.5 to 47.5%, whereas the activity of surface-displayed proteins ranged from 52.5 to 55.8%, indicating that approximately half of the total applied activity was associated with surface-displayed β-xylosidase. However, the hydrolytic activity of the surface-displayed β-xylosidase without the linker peptide only exhibited 7.8% and 9.4% activity (for CshA-Xyl and Xyl-CshA, respectively), which corresponded to only 49.8 and 64.3 U/g dry BLP weight, respectively. By contrast, BLP displaying β-xylosidase with the flexible (GGGGS)_3_ linker peptide (CshA-L-Xyl and Xyl-L-CshA) showed higher activity (51.9% and 52.6% respectively), corresponding to 340.0 and 353.7 U/g dry BLP weight, respectively. Consequently, the activity retention values of surface-displayed β-xylosidase were substantially higher at 96.7% and 98.3% than those without the linker peptide at only 14.4% and 17.6%. These results strongly suggest that the addition of a flexible linker peptide is crucial for the functionality of surface-displayed β-xylosidase.

**Table 4:** Enzyme activities of surface-displayed β-xylosidases

Displayed fusion protein on BLP	Residual activity of unbound enzyme^1^, %	Surface display yield^2^, %	Activity on cell surface		Activity retention^5^, %
			%^3^	U/g dry BLP weight^4^	
CshA-Xyl	47.5 ± 0.6	52.5 ± 0.6	7.8 ± 0.1	49.8 ± 0.8	14.4 ± 0.1
Xyl-CshA	44.5 ± 0.7	55.5 ± 0.7	9.4 ± 0.1	64.3 ± 0.9	17.6 ± 0.1
CshA-L-Xyl	46.3 ± 0.4	53.7 ± 0.4	51.9 ± 0.4	340.0 ± 6.1	96.7 ± 0.2
Xyl-L-CshA	45.2 ± 0.7	55.8 ± 0.7	52.6 ± 0.9	353.7 ± 2.6	98.3 ± 0.4

^1^The residual activity of the unbound enzymes was defined as the percentage of the activity of the unbound enzyme after surface display relative to total applied activity

^2^The surface display yield was calculated by subtracting the activity of the unbound enzyme to the total applied activity

^3^The activity on cell surface (%) was calculated as the proportion of the activity of surface displayed enzyme to the total applied activity

^4^The activity on BLP surface was calculated based on the units of enzymes (U) per dry weight of BLP (g)

^5^The activity retention was calculated as the proportion of the activity of surface displayed enzyme to the surface display yield

Values are reported as mean ± standard deviation of triplicate experiments

### Hydrolysis of xylo-oligosaccharides using surface-displayed β-xylosidase

**Fig. 4:**
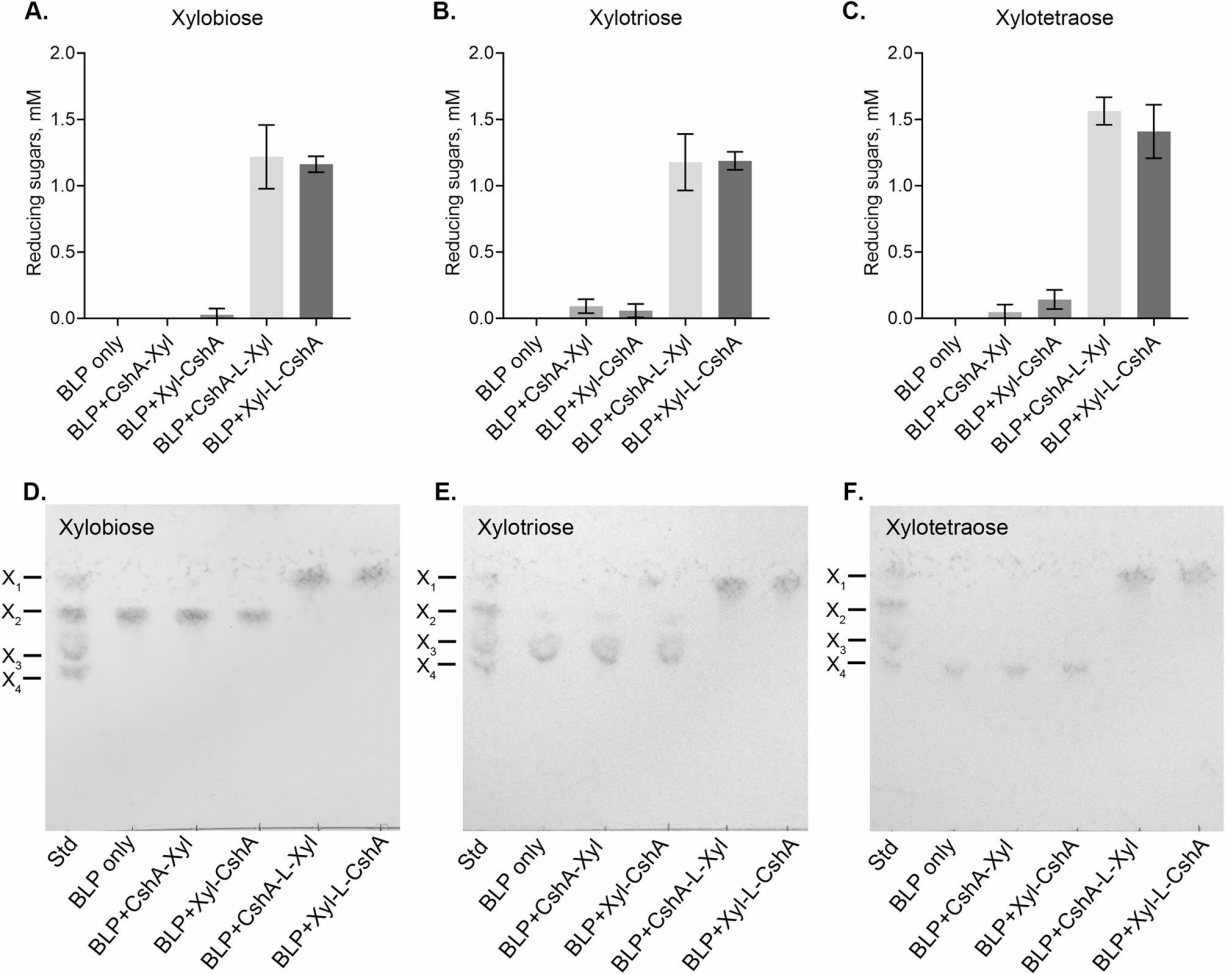
Hydrolysis of xylo-oligosaccharides (XOS) by bacteria-like particles (BLP)-displaying β-xylosidase. Release of reducing sugars equivalent to xylose (in mM) by surface-displayed β-xylosidase from 2 mM xylobiose (**A**), xylotriose (**B**), and xylotetraose (**C**). Thin-layer chromatography analysis of the degradation of XOS using surface-displayed β-xylosidase: xylobiose (**D**), xylotriose (**E**), and xylotetraose (**F**). The enzyme assays were performed in 50 mM sodium phosphate buffer (pH 7.0), at 35 °C, for 5 min. Assays were performed in triplicates. Abbreviations: Std, standard; X_1_, xylose; X_2_, xylobiose; X_3_, xylotriose; and X_4_, xylotetraose

To examine the activity of surface-displayed β-xylosidase towards XOS, enzyme assays using different XOS substrates (xylobiose, xylotriose, and xylotetraose) were performed. Surface-displayed β-xylosidase without the flexible linker peptide (CshA-Xyl and Xyl-CshA) demonstrated weak activity against xylobiose, xylotriose, and xylotetraose (Fig. 4A–C). Compared to surface-displayed β-xylosidase without the linker peptide, CshA-L-Xyl and Xyl-L-CshA (i.e., with linker peptide) had higher catalytic activity towards all XOS substrates (Fig. 4A–C). These findings were consistent with the results of TLC analyses, wherein higher yields of xylose monosaccharides from XOS hydrolysis were achieved using surface-displayed β-xylosidase with peptide linker (Fig. 4D–F).

### Reusability of surface-displayed β-xylosidase

**Fig. 5:**
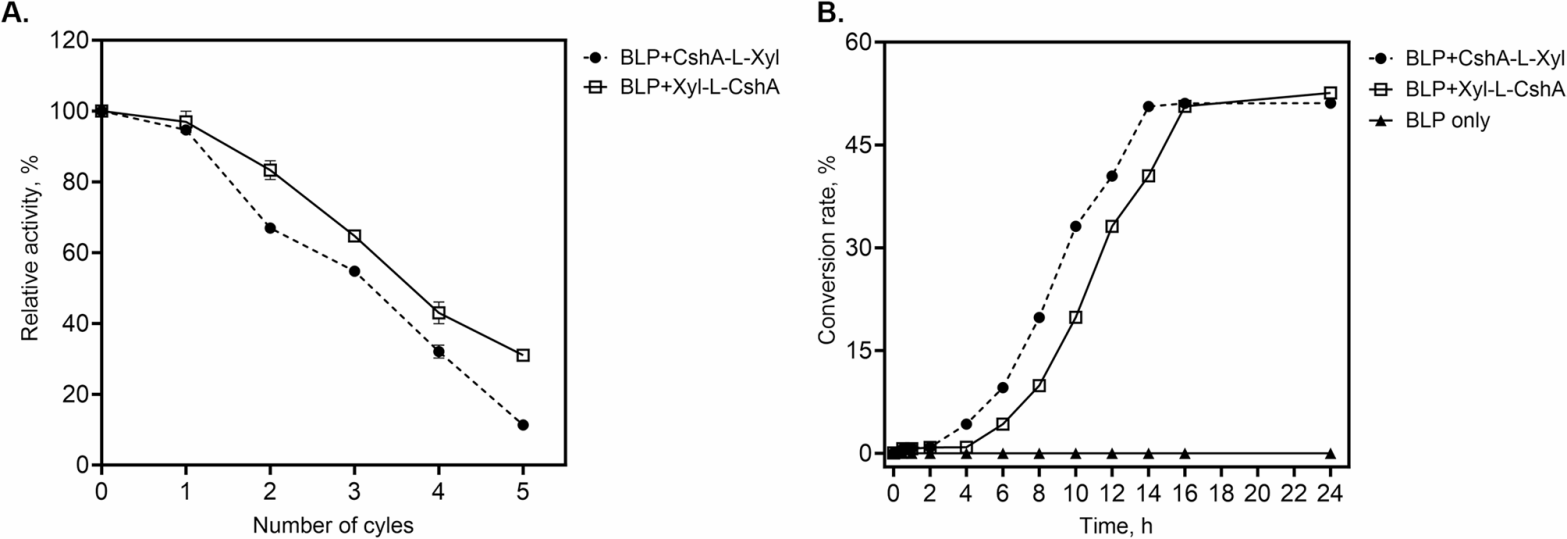
Reusability of bacteria-like particles (BLP)-displaying β-xylosidase and the time course xylose production. The activity of surface-displayed β-xylosidase with N-terminal anchor (BLP + CshA-L-Xyl) or C-terminal anchor (BLP + Xyl-L-CshA) after several bioconversion cycles (**A**). A washing step in between the cycles was performed before the addition of fresh substrate. Time-course production of xylose from 30 g/L beechwood xylan using non-displaying BLP or BLP displaying either CshA-L-Xyl or Xyl-L-CshA (**B**). The total concentration of surface-displayed β-xylosidase per mL of reaction was 0.5 mg. The reactions were performed in 50 mM sodium phosphate buffer (pH 7.0), at 35 °C. Data are reported as the mean ± standard deviation of triplicate experiments. Error bars smaller than the symbol are not shown

The reusability of surface-displayed β-xylosidase was also investigated in this study by measuring β-xylosidase activity upon repeated bioconversion cycles (Fig. 5A). Only the surface-displayed β-xylosidase with linker peptides, namely CshA-L-Xyl and Xyl-L-CshA, were examined for their reusability because these surface-displayed enzymes showed high activity upon immobilisation. After the first cycle, the activities of CshA-L-Xyl and Xyl-L-CshA were retained at 94% and 97%, respectively. At the end of the second cycle, the hydrolytic activity of CshA-L-Xyl decreased by 36%, whereas that of Xyl-L-CshA decreased by only 16%. After the third conversion cycle, the activity was further reduced to 55% and 66% for CshA-L-Xyl and Xyl-L-CshA, respectively. After the fourth conversion cycle, the surface-displayed β-xylosidase with the N-terminal anchor retained 32% of its activity, whereas the enzyme with the C-terminal anchor still retained 43% activity. After the last bioconversion cycle, CshA-L-Xyl and Xyl-L-CshA retained 11% and 31% activity, respectively. This demonstrates that BLP displaying β-xylosidase can be reused after several conversion cycles.

### Production of xylose from Beechwood Xylan using surface-displayed β-xylosidase

To investigate the ability of the surface-displayed β-xylosidase to degrade xylan hemicellulose and produce xylose, the time-dependent saccharification of beechwood xylan using BLP displaying β-xylosidase was performed and monitored using HPLC (Fig. 5B). No detectable xylose formation was observed in the reaction containing non-displaying BLP. In contrast, the maximum yields of xylose from 30 g/L beechwood xylan for surface-displayed CshA-L-Xyl and Xyl-L-CshA were 51% and 53%, respectively, which were achieved after 14–16 h of the reaction (Fig. 5B). This demonstrates the ability of surface-displayed β-xylosidase to degrade xylan hemicellulose and produce industrially important xylose.

## Discussion

Surface display using LAB is a promising approach for immobilising foreign proteins on the surfaces of food-grade bacteria (Lee et al. 2003; Zadravec et al. 2015; Michon et al. 2016; Mao et al. 2016; Park 2020). In recent years, research on non-GMO surface display has garnered attention, particularly in the development of vaccines, wherein significant progress has been made (Zadravec et al. 2015; Mao et al. 2016; Pereira et al. 2022). In addition to vaccine development, non-GMO surface display using LAB is now being explored to immobilise industrially and therapeutically important enzymes. For instance, Pham and colleagues (2019a) successfully displayed two β-galactosidases on the surfaces of different LAB strains, such as *Lp. plantarum*, *Lactobacillus delbrueckii* subsp. *bulgaricus*,* Lacticaseibacillus casei*, and *Lactobacillus helveticus*, using the surface anchor LysM. In another study, Nguyen et al. (2024) surface-displayed mannanase and chitosanase on *Lp. plantarum* using single or double LysM domains. Similarly, the LysM domain was used by Hu et al. (2010) to display β-galactosidase on different LAB strains. The cell-anchoring domain of the SH3 type 5 motif was successfully used to non-recombinantly display superoxide dismutase on the surface of *Lm. fermentum* (Tay et al. 2021). Although the LysM motif is a popular anchor in these studies, other surface anchors can be found in LAB, including LPxTG, WxL, S-layer, lipoprotein, N-terminal transmembrane, and non-classical anchors, which provide excellent surface display of the protein of interest on the surface of LAB (Åvall-Jääskeläinen et al. 2002; Glenting et al. 2013; Mao et al. 2016; Gordillo et al. 2020; Mathiesen et al. 2020; Zhou et al. 2023). In our previous study, we identified and characterised a non-classical cell surface anchor, CshA, from *Lp. plantarum*. This novel cell surface anchor putatively binds to the cell wall non-covalently, is influenced by pH, temperature, and ionic salt concentration, and exhibits host-specific display (Vasquez et al. 2022). The CshA-mediated surface display was greatly enhanced when LAB-derived BLP were used as the display matrix. BLP are non-living cells devoid of surface proteins, making them highly suitable for surface display applications (Mao et al. 2016; Zhou et al. 2023). These particles are excellent candidates for the immobilisation of biocatalysts because they are stable, food-grade, easy to use, and economical (Bosma et al. 2006; Mao et al. 2016). In addition, BLP have a high loading capacity and excellent binding stability (Zhou et al. 2023). Previously, Bosma et al. (2006) successfully displayed an α-amylase from *Bacillus licheniformis* on the surface of lactococcal-based BLP, demonstrating its applicability in enzyme immobilisation. Hence, in the current study, CshA was used as the anchor for the immobilisation of β-xylosidase on BLP.

The addition of linker peptides, which allow the separation of proteins with multiple domains, is a popular strategy in fusion protein research (Arai et al. 2001; Chen et al. 2013; Li et al. 2016). Linker peptides are widely used in many biotechnological applications such as protein expression and purification, imaging, immunoassays, and biopharmaceuticals (Chen et al. 2013; Li et al. 2016). Although many linker peptides occur naturally in multidomain proteins, numerous linker peptides have been empirically designed based on the need for fusion proteins (Chen et al. 2013). In surface display studies, the addition of linker peptides allows for a more successful display on the cell surface without compromising the activity of the immobilised enzyme (Hinc et al. 2013; Michon et al. 2016; Su et al. 2023). In the current study, we successfully constructed and expressed four anchor-xylosidase fusion proteins in *E. coli*: CshA-Xyl, Xyl-CshA, CshA-L-Xyl, and Xyl-L-CshA. For the anchor-xylosidase fusion proteins CshA-L-Xyl and Xyl-L-CshA, a linker peptide consisting of three repeats of glycine and serine residues (GGGGS)_3_ was added between the anchor and the enzyme. This provides adequate separation and flexibility for the anchor and the β-xylosidase domains of the fusion protein (Chen et al. 2013; Hinc et al. 2013). We found that all fusion proteins were active towards the artificial substrate pNPX, and neither the position of the anchor nor the addition of the linker peptides disrupted the activity of the free fusion proteins. Proper enzyme-substrate interaction is necessary for successful hydrolysis of the glycosyl linkages at the catalytic site (Manzoor et al. 2023). This indicates that, as unbound proteins, the enzyme-substrate interaction was not interrupted by the addition of linker peptide or anchor (regardless of the position). Moreover, all anchor-xylosidase fusion proteins were successfully displayed on the surface of BLP, as revealed by immunofluorescence microscopy and flow cytometry. Notably, neither the position of the anchor nor the addition of linker peptides had substantial effects on CshA-mediated display on the surface of BLP, suggesting that the binding of the anchor to the peptidoglycan layer of the BLP was not interrupted by the addition of β-xylosidase at either position. However, the functional activities of β-xylosidase without the linker peptide were considerably reduced by approximately 6-fold upon surface display on BLP than those with the linker peptide. Furthermore, β-xylosidase fusion proteins with linker peptide showed excellent retention of their hydrolytic activities versus β-xylosidases displayed without the linker peptide. The loss of functional activity of the anchor-xylosidase fusion protein without the linker peptide upon surface display may be due to a lack of enzyme mobility, thereby hindering substrate access to the catalytic pocket. Conformational changes in the enzyme upon immobilisation are also possible, leading to poor hydrolytic activity (Purich 2010; Li et al. 2025). These observations underscore the importance of linker peptides for maintaining the activity of surface-displayed biocatalysts.

BLP displaying β-xylosidase exhibited an average display yield of 54.3%. The observed display yield in the current study is higher than the LysM-mediated display yield of chitosanase (41.6–44.6% immobilisation yield), mannanase (41.7–48.7% immobilisation yield), or β-galactosidases (6.5–31.9% immobilisation yield). Nevertheless, it must be noted that the surface display yield is dictated by the binding capacity of the cells/particles, the size of the protein being displayed, and the anchor used, all of which may explain the differences in immobilisation yields among these studies (Gordillo et al. 2020; Raya-Tonetti et al. 2021; Tay et al. 2021; Vasquez et al. 2022). Thus, the retained activity of the displayed biocatalyst could be a better measure of the immobilisation success (Boudrant et al. 2020). Herein, the surface-displayed β-xylosidase (with a linker) demonstrated exceptional activity retention (97.5%), indicating that almost all of the displayed β-xylosidase molecules were catalytically active. In other studies, the surface-displayed chitosanase and mannanase activities on *Lp. plantarum* showed an average activity retention of 86.4% and 74.2% (Nguyen et al. 2024), whereas surface-displayed LacZL-type β-galactosidase had activity retention of 58.2% across different LAB strains as display hosts (Pham et al. 2019b). Moreover, spores displaying β-xylosidase demonstrated 65% activity retention (Mattossovich et al. 2017), and CotG-mediated surface-displayed β-galactosidase and L-arabinose isomerase retained 61% and 89% of their catalytic activities, respectively, after immobilisation (Kwon et al. 2007; Guo et al. 2018). Moreover, both surface-displayed β-xylosidase with linker peptide showed catalytic activity towards XOS with varying degrees of polymerisation, specifically xylobiose, xylotriose, and xylotetraose. Moreover, BLP displaying β-xylosidase also demonstrated their ability to degrade beechwood xylan to produce xylose monosaccharide. At the end of the bioconversion reaction, surface-displayed β-xylosidase were able to convert about 50% (about 15 g/L) of the original beechwood xylan substrate to xylose monosaccharides. Although the bioconversion was not complete, it was expected since complete degradation of xylan hemicellulose requires the concerted activities of many enzymes, such as xylanases. The remaining un-hydrolysed substrate must contain glycosyl linkages that cannot be hydrolysed by β-xylosidase used herein. This shows that β-xylosidase immobilised on the surface of BLP is comparable with previously reported immobilised biocatalysts and is active towards xylan hemicellulose, which demonstrates its potential applicability for industrial use.

Finally, BLP displaying β-xylosidase demonstrated comparable reusability to other surface-displayed enzymes. Notably, BLP displaying Xyl-L-CshA showed better reusability as this C-terminally anchored β-xylosidase retained 2.8-fold higher activity after the fifth conversion cycle than N-terminally anchored β-xylosidase. This suggests that the C-terminal anchoring of β-xylosidase is more stable than its N-terminal counterpart. It has been shown that immobilisation improves the stability of the enzyme, thus enhancing its reusability (Boudrant et al. 2020; Maghraby et al. 2023). In addition, the C-terminal position of the anchor may have allowed the formation of a more stable oligomeric state of the β-xylosidase (Corradini et al. 2021), thereby maintaining its activity after several bioconversion cycles. The reusability of BLP displaying β-xylosidase is consistent with that of other biocatalysts immobilised on bacterial or spore surfaces, with reusability ranging from 4 to 5 conversion cycles (Mattossovich et al. 2017; Guo et al. 2018; Pham et al. 2019b, 2020; Nguyen et al. 2024).

The present study has a few limitations that should be further explored in the future. The influence of other types of peptide linkers, such as rigid linkers, on the display performance and activity of the biocatalysts should be examined. Furthermore, the synergistic activity of surface-displayed β-xylosidase with xylanase could be investigated for the degradation of other xylan hemicellulose substrates. Finally, the reusability of BLP displaying β-xylosidase warrants further improvement akin to that described in other reports (Morana et al. 2006; Guerfali et al. 2009; Terrasan et al. 2017; Corradini et al. 2021; Romero et al. 2023).

## Conclusion

This study demonstrates that the β-xylosidase from *Lm. fermentum* can be successfully displayed on the surface of LAB-derived BLP using the novel anchor CshA. The BLP surface displaying the β-xylosidase showed activity towards pNPX, XOS, and beechwood xylan. Moreover, the activity of surface-displayed β-xylosidase was influenced by the presence of linker peptides. BLP displaying β-xylosidase had high surface display yield and activity retention, comparable to other immobilised biocatalysts. Furthermore, BLP displaying β-xylosidase demonstrated stability and reusability after several bioconversion cycles. To our knowledge, this study is the first report to demonstrate the application of non-GMO surface display to immobilise a β-xylosidase for xylan degradation. Furthermore, this study provides proof-of-concept for the use of non-GMO, food-grade surface-displayed β-xylosidase in the saccharification of lignocellulosic biomass, with potential industrial uses. Future research should aim to investigate process optimisation, including enhanced enzyme retention and multi-enzyme co-immobilisation, to validate and extend these findings. By addressing these challenges, this study will establish the foundation for cost-effective and eco-friendly biocatalyst applications in biofuel production and other industrial bioprocesses.

## Data Availability

The datasets presented in this study can be found in online repositories. The name of the repository and accession number can be found in the article.

## Abbreviations

BLP: Bacteria-like particles
GGGGS_3_: Three repeats of glycine-serine residues
GH: Glycoside hydrolase
GMO: Genetically modified organism
GRAS: Generally regarded as safe
HPLC: High-performance liquid chromatography
LAB: Lactic acid bacteria
PBS: Phosphate-buffered saline
PCR: Polymerase chain reaction
pNPX: *p*-nitrophenyl-β-D-xylopyranoside
TLC: Thin layer chromatography
XOS: Xylo-oligosaccharides
